# Light as stress factor to plant roots – case of root halotropism

**DOI:** 10.3389/fpls.2014.00718

**Published:** 2014-12-12

**Authors:** Ken Yokawa, Rossella Fasano, Tomoko Kagenishi, František Baluška

**Affiliations:** ^1^Department of Plant Cell Biology, Institute of Cellular and Molecular Botany, University of BonnBonn, Germany; ^2^Department of Biological Sciences, Tokyo Metropolitan UniversityTokyo, Japan; ^3^Department of Pharmacy, University of SalernoFisciano, Italy

**Keywords:** root, light response, plant hormones, reactive oxygen species, root tropism

## Abstract

Despite growing underground, largely in darkness, roots emerge to be very sensitive to light. Recently, several important papers have been published which reveal that plant roots not only express all known light receptors but also that their growth, physiology and adaptive stress responses are light-sensitive. In *Arabidopsis*, illumination of roots speeds-up root growth via reactive oxygen species-mediated and F-actin dependent process. On the other hand, keeping *Arabidopsis* roots in darkness alters F-actin distribution, polar localization of PIN proteins as well as polar transport of auxin. Several signaling components activated by phytohormones are overlapping with light-related signaling cascade. We demonstrated that the sensitivity of roots to salinity is altered in the light-grown *Arabidopsis* roots. Particularly, light-exposed roots are less effective in their salt-avoidance behavior known as root halotropism. Here we discuss these new aspects of light-mediated root behavior from cellular, physiological and evolutionary perspectives.

## LIGHT AS IMPORTANT ENVIRONMENTAL FACTOR FOR ROOTS

In nature, sessile plants have to respond to diurnal change in the light environment. One of main roles of light in plant’s life is to provide energy for photosynthesis and for the regulation of plant development at different stages such as seed germination, vegetative growth, tropisms and flowering. It is known that plant photoreceptors and related light-sensitive signaling molecules participate in the regulation of physiological conditions and morphological plasticity in response to the light environment. Darwin (1879) has discovered negative phototropism of plant roots. One year later, Francis Darwin and his father, Charles Darwin, published the book, “The Power of Movements in Plants.” They described both root and shoot tropisms. In addition, they also proposed that some form of long-distance signaling connect the sensory organ apices with the actively growing basal parts (Darwin, 1879, 1880). Since then, dedicated research work in plant physiology has discovered the long-distance signaling molecule, auxin, resulting in insights into plant photoreception. This directional growth response to incoming light is called phototropism. Positive phototropism, observed in shoots, is growth toward a light source; whereas negative phototropism, seen in roots, is bending away from the light source. We demonstrated that short (10 s), but strong (the photon flux was 82 μmol m^-2^ s^-1^), blue light illumination of *Arabidopsis* roots induces the immediate generation of reactive oxygen species (ROS) in root apex region, resulting in rapid increase of the root growth rate (Yokawa et al., 2011, 2013). This active response of light-stimulated root growth is termed escape tropism (Xu et al., 2013; Yokawa et al., 2013; for maize roots see Burbach et al., 2012). This tropism would allow *Arabidopsis* roots to escape from unfavorable light conditions if growing outside of our laboratories in the nature.

## PHOTORECEPTORS IN ROOTS

It has been shown that *Arabidopsis* plant expresses 14 photoreceptors, most of which are also present in roots (Briggs and Lin, 2012; Jeong and Choi, 2013; Briggs, 2014). Roots grow in the dark soil to anchor the plant and to absorb mineral nutrients and water. It has been reported that light can penetrate less than several millimeters due to the rather high absorbance of soil (Woolley and Stoller, 1978). Nevertheless, small cracks or mechanical impacts can often happen which allows light to penetrate deeper. For instance, roots may be exposed to light due to sudden temperature changes, earthquake, heavy rain, wind, and so on. In addition, it is very important for emerging radicle to increase the root growth rate shortly after seed germination on the ground. It was necessary to evolve the ability of roots to respond to environmental light when the first flowering plants with modern root system emerged in land plant evolution. In the next section, intriguing interplays between phytohormones and light-related signaling pathways will be discussed.

## FROM ACTIN CYTOSKELETON, VIA PIN2 RECYCLING, TO SALT AVOIDANCE TROPISMS OF ROOTS

At the cellular level, it was reported that PIN2 proteins (PIN-FORMED 2; auxin eﬄux carrier) in root apices respond to the light environment (Laxmi et al., 2008). Wan et al. (2012) demonstrated that the basipetal (shootward) PIN2-based polar auxin transport is subject to blue light control, which regulates the negative phototropism of *Arabidopsis* roots (Wan et al., 2012). Moreover, Dyachok et al. (2011) reported that light-activated COP1, E3 ubiquitin ligase, promotes actin polymerization and F-actin bundling, through regulation of the downstream ARP2/3-SCAR pathway in root cells. It results in increased root growth under the illuminated conditions (Dyachok et al., 2011). It was also reported that light controls bundling of F-actin in maize coleoptiles (Waller and Nick, 1997), changing sensitivity of cells to auxin, which is feeding back to control F-actin as well as cell growth (Nick et al., 2009). The interplays between F-actin and polar auxin transport mediated by endocytic vesicle recycling, especially in the transition zone of root apex, control root tropisms (Baluška et al., 1996, 2004, 2005, 2010; Baluška and Mancuso, 2013). Interestingly, precursor of endogenous auxin, indole-3-acetaldehyde (IAAld), is produced non-enzymatically *in vitro* by illumination of tryptophan in the presence of flavin which is abundant in living plant cells (Koshiba et al., 1993). Recently, we have proposed close links between the redox status and auxin (IAA) biosynthesis in plants (Yokawa et al., 2014). Taken together, it is obvious that roots are extraordinarily sensitive to light exposure due to their inherent evolutionary optimization for the underground life. Therefore, it is not surprising that illuminated roots of young *Arabidopsis* seedlings enhance their growth with the concomitant phototropism.

A few yeas ago, salt-stressed roots of *Arabidopsis* have been shown to alter root growth direction in order to avoid high salt areas via so-called salt avoidance tropism (Li and Zhang, 2008; Sun et al., 2008). This active root tropism requires ion gradient sensing pathway which would then control the PIN2 abundance, recycling and degradation (Li and Zhang, 2008; Sun et al., 2008). This unique *Arabidopsis* root behavior was linked to phospholipase D Zeta2 (PLDζ2) activity which stimulates clathrin-mediated endocytosis of PIN2, and this tropism was also termed root halotropism (Galvan-Ampudia et al., 2013; Rosquete and Kleine-Vehn, 2013; Pierik and Testerink, 2014). Interestingly, similarly, as in halotropism, PLDζ2 is involved in root hydrotropism through the PIN2-mediated suppression of root gravitropism (Taniguchi et al., 2010). Moreover, PLDζ2 is crucial for brefeldin A-sensitive endocytic recycling driving PIN2 recycling (Li and Xue, 2007) and polar auxin transport in the transition zone (Mancuso et al., 2007). Because PIN2 is crucial in this respect, as well as in adaptive responses of roots to light (Laxmi et al., 2008; Sassi et al., 2012; Wan et al., 2012), it is very important to test whether light condition affect the response of *Arabidopsis* roots to the salt stress.

## PLANT HORMONES ARE INTEGRATED WITH LIGHT SIGNALING PATHWAYS

**FIGURE 1:**
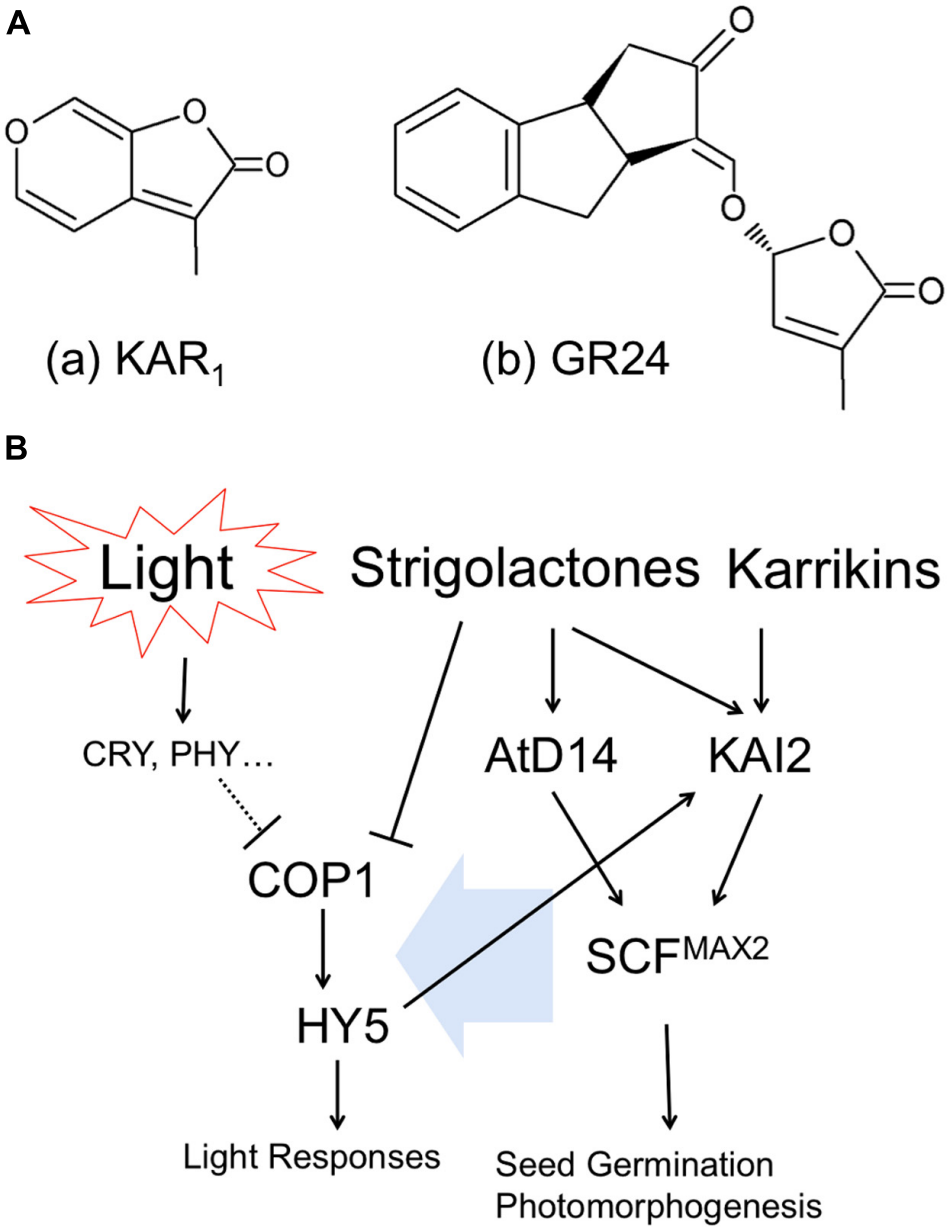
**Cross talk between butenolide plant hormones and light signaling pathways. (A)** Chemical structures of (a) Karrikin1, KAR_1_ and (b) Strigolactone analog, GR24. **(B)** Interaction of hormonal and light signaling pathways.

Karrikin molecule was isolated from the smoke of combusted plant materials and found to potently stimulate the germination of plant seeds (Flematti et al., 2004). This compound was identified as 3-methyl-2H-furo[2,3-c]pyran-2-one, karrikin-1 or karrikinolide-1 (KAR_1_) shown in **Figure 1A**-a, and another analogs of karrikins (KAR_2_-KAR_6_) commonly possess butenolide structure. The *Arabidopsis* mutant *kai2* lacking *KAI2* genes insensitive to KAR_1_ triggered promotion of seed germination. KAI2 is thought to be a putative receptor of karrikins (Nelson et al., 2010; Waters et al., 2012). KAI2 is a member of α/β-hydrolase family and is also known as HYPOSENSITIVE TO LIGHT (HTL). Role of KAI2/HTL in light responses (**Figure 1B**) of roots will be described in the next section.

*Arabidopsis thaliana* AtD14 (ortholog of DWARF14) is a paralog of KAI2/HTL, which belongs to α/β-hydrolase family and plays a role in strigolactone perception (Waters et al., 2012). Strigolactones are synthesized from carotenoids and released into root exudates to promote germination of parasitic weeds (Yoneyama et al., 2007), as well as to stimulate hyphal branching in arbuscular mycorrhizal fungi (Akiyama et al., 2005). Strigolactones act also as a new class of plant hormone that inhibit the growth of axillary buds (Umehara et al., 2008) and alter root architecture (Koltai, 2011). Strigolactones are categorized as a sesquiterpenes lactone and have two moieties of butenolide (shown in **Figure 1A**-b as synthesized analog, GR24). As described above, karrikins have butenolide moieties as well. Therefore, because of the structural similarity of two molecules, karrikin receptor KAI2/HTL can respond to strigolactones. However, in contrast, the proposed strigolactone receptor AtD14 is insensitive to karrikins; indicating that the activity of KAI2/HTL as α/β-hydrolase is more flexible to detect butenolide structure than AtD14, probably due to evolutionary issues (Waters et al., 2012).

It was reported that F-box protein, MORE AXILLARY BRANCHES2 (MAX2), is located in the downstream of both KAI2/HTL and AtD14 and thus necessary for plants to respond to karrikin and strigolactone (Nelson et al., 2010; Waters et al., 2012). MAX2 forms SCF E3 ubiquitin ligase complex (Skp1, Cullin1, F-box), which modulates further downstream transcriptions (Stirnberg et al., 2007; Shen et al., 2012). However, molecular functions of KAI2/HTL or AtD14-mediated SCF^MAX2^ regulation of plant morphogenesis are not known yet. Interestingly in this respect, *max2* mutants are hyper-sensitive to drought and osmotic stress, including high NaCl, mannitol, and glucose (Bu et al., 2014). Furthermore, strigolactones exert positive roles in plant adaptation to drought and salt stress (Ha et al., 2014). We will discuss these newly emerging aspects in the final section.

## LIGHT SIGNAL TRANSDUCTION VIA COP1 AND HY5 IS INTEGRATED WITH HORMONAL SIGNALING

In principle, plants recognize light as specific wavelengths by specific photoreceptors. Activated (excited) photoreceptors convert the light information into physiological signaling in diverse manners. CONSTITUTIVE PHOTOMORPHOGENIC1(COP1), E3 ubiquitin ligase, participates in the light-perceiving signaling cascade via affecting ubiquitination of target proteins. Once the activity of COP1 is inhibited, the downstream bZIP transcription factor, ELONGATED HYPOCOTYL5 (HY5) is freed from ubiquitination by COP1 and starts specific gene transcriptions related to light responses (**Figure 1B** left). COP1 and HY5 control root growth, lateral root formation, root hair tip growth, as well as root touch-responses and gravitropism (Oyama et al., 1997; Ang et al., 1998; Cluis et al., 2004; Datta et al., 2006, 2007; Sibout et al., 2006). Importantly, SALT TOLERANCE HOMOLOG2 (STH2) is interacting partner of COP1 and HY5 which controls roots and their anthocyanin levels (Datta et al., 2007).

Interestingly, exposure of plants to strigolactone inhibits the COP1 activity, suggesting that strigolactones can mimick light perception in plants (Tsuchiya et al., 2010). They also demonstrated that *max2* mutant of rice produces excess of strigolactones, resulting in the inhibition of COP1 and expression of light-responsive genes (Tsuchiya et al., 2010). In addition, it was reported that *max2* mutant is hypersensitive to red, far-red and blue light (Shen et al., 2007). Auxin interaction with the signaling pathway of strigolactones via MAX2 was already reported (Hayward et al., 2009). Finally, the key transcription factor of light signaling HY5 requires strigolactones in order to stimulate *Arabidopsis* seed germination during thermoinhibiton (Toh et al., 2012). Light also induces auxin biosynthesis via photoexcitation of flavins (Koshiba et al., 1993; Yokawa et al., 2014).

Karrikin receptor KAI2 is also known as HTL. KAI2/HTL expression was strongly increased by blue, red and far-red light and HY5 binds to the promoter region of HTL, indicating the expression of KAI2/HTL is regulated in response to light environment (Sun and Ni, 2011). Furthermore, KAI2/HTL is located downstream of HY5 (**Figure 1B**) and induced by light treatment (Sun and Ni, 2011). Meanwhile, it was reported that the treatment of seeds with KAR_1_ (chemical structure is shown in **Figure 1A**-a) improved light responses in germination and early development of seedlings (Nelson et al., 2010). It implies that karrikins can affect the array of gene expression regarding light responses.

Plant roots exposed to salinity stress increase abscisic acid (ABA) and ABA-related gene expressions (Raghavendra et al., 2010). ABA and strigolactones are synthesized from carotenoids and the levels of these two plant hormones are affecting each other (López-Ráez et al., 2010). Salt stress increases strigolactone levels in roots which then promote arbuscular mycorrhizal symbiosis, resulting in changing ABA contents and alleviating stress response (Aroca et al., 2013). Strigolactones, ABA and cytokinin signaling pathways are integrated to allow plants to cope with high salinity environment very effectively (Ha et al., 2014). Many players of the phytohormone signaling pathways are overlapping and integrating with light-response pathways.

Taken together, these findings suggest that hormonal and light signaling pathway utilizes same junction for plant morphogenesis as well as for adaptation to abiotic stresses. The signaling pathways proposed (**Figure 1B**) are highly integrated, interacting with each other and, obviously, they represent just a ‘tip of the iceberg.’ In the case of roots, we should consider light solely as information (not as source of energy) into our careful consideration for our understanding of plant photomorphogenesis. In the next session, we are discussing how the root sensitivity to salinity is altered via exposure of roots to light.

## UV LIGHT AND UVR8 IN DROUGHT AND SALINITY RESPONSES OF ROOTS

It has been reported that the reduction of plant growth under water deficit is driven by the UV-B photoreceptor UV RESISTANCE LOCUS 8 (UVR8; Kliebenstein et al., 2002; Brown et al., 2005; Favory et al., 2009; Fasano et al., 2014). Fasano et al. (2014) previously shown that the *UVR8* gene complements the osmosensitive yeast mutant *mpk1 ppz1*. The expression of *UVR8* was found upregulated in *Arabidopsis* plants grown under salt or osmotic stress conditions. Furthermore, the ectopic expression of *UVR8* causes pleiotropic effects on plant growth, such as a general reduction of plant organ size, leaves with smaller cells, reduced root growth, and the accumulation of flavonoids. This suggests that the UV-B morphogenic responses are enhanced in the *UVR8*-overexpressing plants grown under low levels of UV-B light. The growth defects of the *UVR8*-overexpressing plants are even more severe under osmotic and salt stress. In contrast, the inactivation of *UVR8* expression does not affect shoot or root growth under standard or mild drought stress conditions. Thus, the hypersensitive response to osmotic stress of the *35SUVR8* plants is strictly UVR8-dependent (Fasano et al., 2014).

There are extensive evidences that osmotic stress as well as UV-B light impacts are more prominent on shoots than on roots. Under mild osmotic stress, roots continue to grow (Bartels and Sunkar, 2005; Pardo, 2010; Werner et al., 2010). Moreover, enhanced root growth was observed in transgenic plants with a higher drought or salt-stress tolerance (Bartels and Sunkar, 2005). Whereas the impact of osmotic stress on root development is well known (recently reviewed in Liu et al., 2014), effects of UV-B on root growth are poorly understood. In general UV-B reduces primary root growth of *Arabidopsis* seedling (Kim et al., 1998; Tong et al., 2008). In the adult plant, increased allocation of biomass to roots has been reported to occur under UV-B stress (Bussell et al., 2012).

**FIGURE 2:**
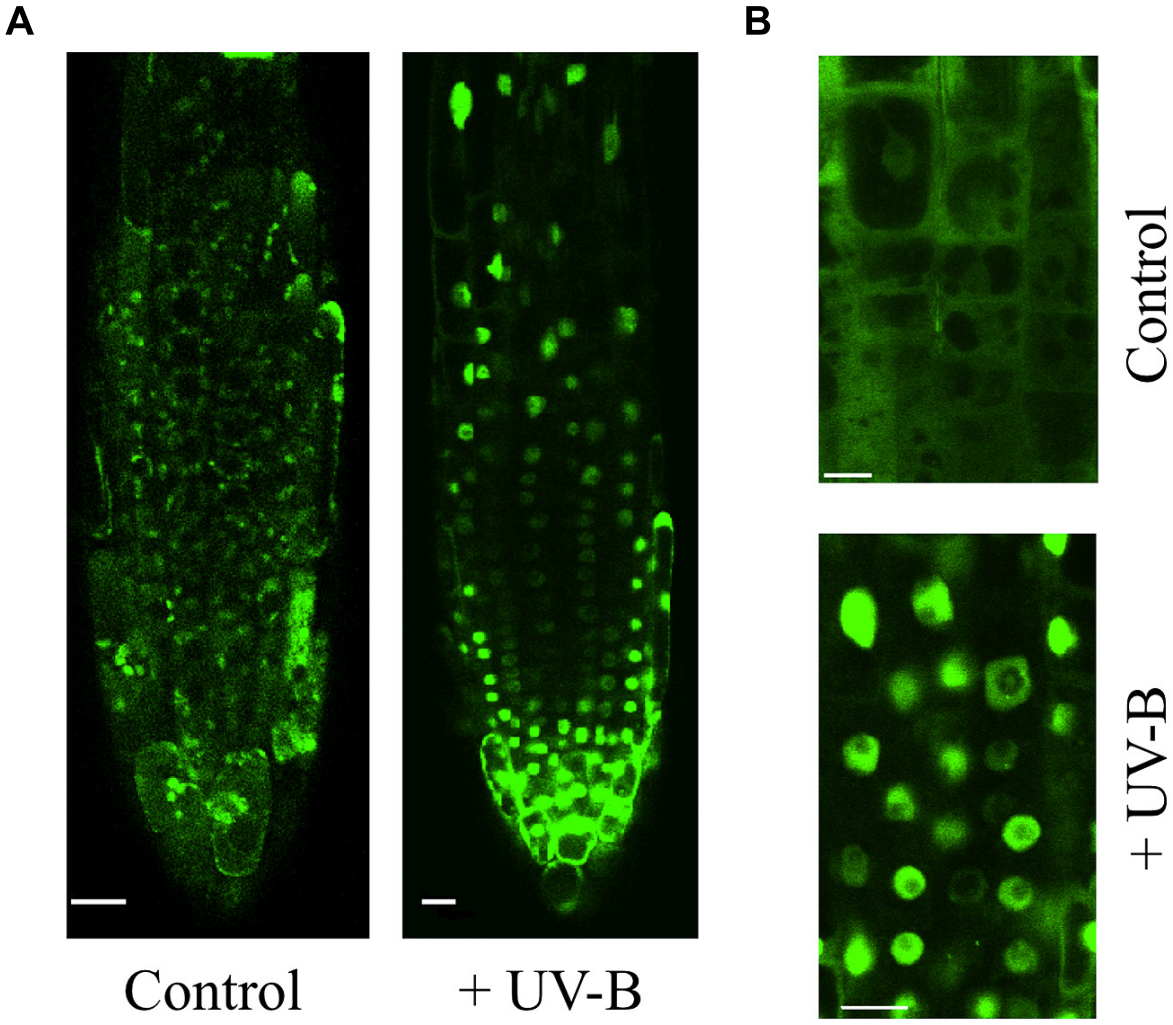
**Relocation of UVR8::GFP to nuclei in *Arabidopsis* root cells. (A)** Root apex region. **(B)** Magnified view of cells in the transition zone. Bar indicates 10 μm. *Arabidopsis* roots were exposed to 1 h UV-B treatment (3.9 μmol m^-2^ s^-1^).

Development of a larger root system is considered as a drought-avoidance strategy that plants adopt to improve the uptake of water and nutrients when their availability in the soil is limited (Fukai and Cooper, 1995; Liao et al., 2001; Sharp et al., 2004; Pierik and Testerink, 2014). The UV-B photoreceptor UVR8 is expressed in roots of wild type plants (Rizzini et al., 2011; reviewed in Yokawa and Baluška, 2014). As shown in **Figure 2**, UVR8::GFP is expressing in *Arabidopsis* roots and is transported into nuclei upon irradiation of roots with UV-B. Over-expression of UVR8 greatly reduces root growth under light exposure and aggravates their weaker performance under the osmotic stress (Fasano et al., 2014). In comparison to the control plants, the primary root and lateral root densities were 13 and 60%, respectively. This indicates that auxin-dependent lateral root growth was most hampered. Indeed the root-phenotypes of the *35S-UVR8* plants are reminiscent that one of auxin mutant (Zhao, 2010). The analysis of lateral roots showed that the number of lateral root primordia and emerged lateral roots of the *35S-UVR8* plants was 12 and 68%, respectively, reduced in comparison to the control plants (Fasano et al., 2014). Flavonoids accumulation was found to be 2.2-fold increased in the root of the *UVR8*-over-expressing plants, whereas the content of IAA-conjugates showed a tendency to decrease (Fasano et al., 2014). Thus, the defects in cell expansion of the *35S-UVR8* roots could be associated to the increased levels of flavonoids which, in turn, alter polar auxin transport and/or auxin homeostasis (e.g., Santelia et al., 2008).

Flavonoids are synthesized in roots, regulating root branching, gravitropism, and stress adaptation (Brown et al., 2001; Buer and Muday, 2004; Agati et al., 2011; Emiliani et al., 2013). Flavonoid concentrations are significantly higher in combined stress-treated plants than in those treated with UV-B alone (Hughes et al., 2010; Comont et al., 2012). Together, all these reports suggest that *UVR8*, via the control of flavonoid accumulation, might be a common intermediate in light and hormone signaling pathways to regulate root growth and development under abiotic stress challenges. Besides UV-B, drought and salinity have also been associated with anthocyanin accumulations in various tissues including roots (Agati et al., 2011; Emiliani et al., 2013; Meng, 2014). Importantly in this respect, the light-induced stimulation of *Arabidopsis* root growth is mediated via the COP1-mediated accumulation of anthocyanins (Meng, 2014).

## IMPACT OF LIGHT ON SALINITY AVOIDANCE VIA ROOT HALOTROPISM

Although root apices growing in soil are known to be at a front line that can be exposed to salt stress, an impact of light on modulation of the sensitivity of roots to salinity has not been considered to date. Importantly, most experiments using the laboratory grown *Arabidopsis* seedlings are using roots exposed to light, although root apices outside in the nature are mostly in darkness.

**FIGURE 3:**
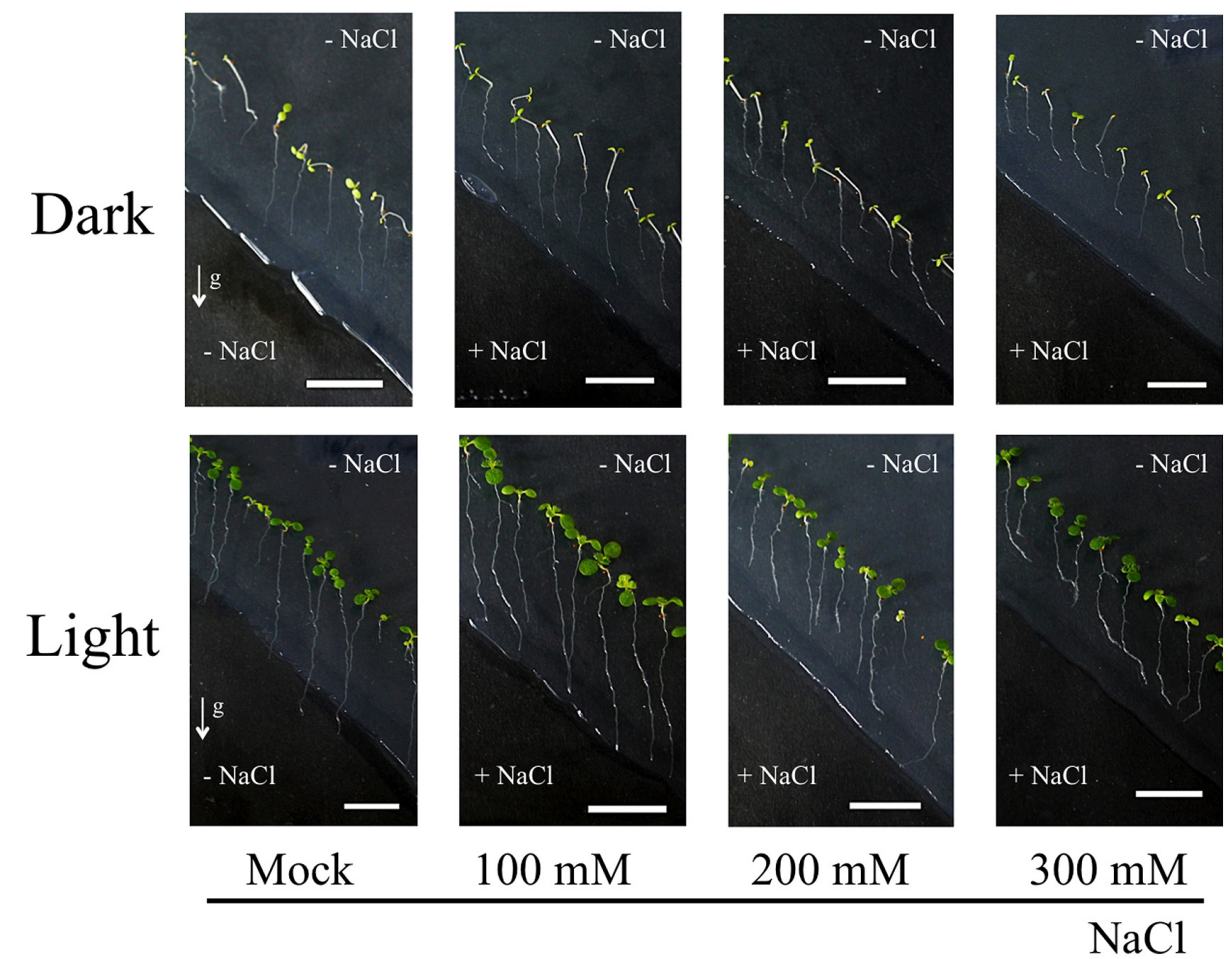
**Light environment affects sensitivity of *Arabidopsis* roots to salinity.** Seedlings of Col-0 were firstly grown on 1/2 MS media for 5 days. Bottom part of agar was removed diagonally and new 1/2 MS media containing NaCl was added. Petri dishes were placed either in light or dark condition for 3 days. Roots growing on agar plates in dark/light conditions with 0, 100, 200, and 300 mM NaCl media in the bottom half of Petri dishes. The method used for imposing the root halotropism is following exactly the protocol as published by Galvan-Ampudia et al. (2013). White arrows indicate the gravity vector. All seedlings were grown in vertical position. Bar indicates 1 cm.

As shown in the **Figure 3**, we have demonstrated that light exposure affects the root response to the salinity stress. Columbia WT seedlings were grown on agar plates containing NaCl only at their bottom parts. With this system, root tropism responses to salinity, halotropism, can be observed. Our methods followed the protocol described in (Galvan-Ampudia et al., 2013; Rosquete and Kleine-Vehn, 2013). After replacing bottom part of agar with the NaCl-agar, plastic dishes were covered with aluminum foil to allow dark treatment. Light/dark period was 16 h/8 h and light intensity was about the 120 μmol m^-2^ s^-1^, comparable to conditions in Galvan-Ampudia et al. (2013). Both the dark- and light-grown roots grew straight to the boundary of two agar media in control experiments with 0 mM NaCl (**Figure 3**). On the other hand, roots growing in darkness have higher sensitivity to NaCl than that roots exposed to light (**Table 1**). Dark-grown roots showed halotropism by avoiding salt-enriched agar areas at all concentrations of NaCl (**Figure 3**; **Table 1**). At the 100 mM concentration of NaCl, light-grown roots kept growing into the salty part of agar across the borderline between two agar parts even after several days. This suggests that light either prevents roots to accomplish the halotropism or that light lowers the sensitivity of *Arabidopsis* roots to perceive a gradient of salinity. The fact that light grown roots performed halotropism at higher concentrations of NaCl (**Table 1**) suggests that light interferes with the elusive root’s ability to sense the Na^+^ gradients (Maathuis, 2014). As root apices of roots growing outside in the nature are typically located deep in the soil in darkness, it can be expected that their halotropism is more efficient than for roots of laboratory grown seedlings.

**Table 1:** Halotropic bendings of *Arabidopsis* roots grown in different light conditions.

	Mock	100 mM	200 mM	300 mM
Dark	0% (*n* = 25)	26% (*n* = 23)	90% (*n* = 30)	92.3% (*n* = 26)
Light	0% (*n* = 29)	18.5% (*n* = 27)	16.7% (*n* = 30)	46.4% (*n* = 28)

*Roots have been considered halotropic when scored root apex angles are greater than 45° with respect of the gravity vector (= same angle with the boundary of agar).*

## LIGHT-INDUCED ROOT GROWTH IS EXPENSIVE AND ALTERS PHYSIOLOGY AND MORPHOLOGY OF WHOLE SEEDLINGS

**FIGURE 4:**
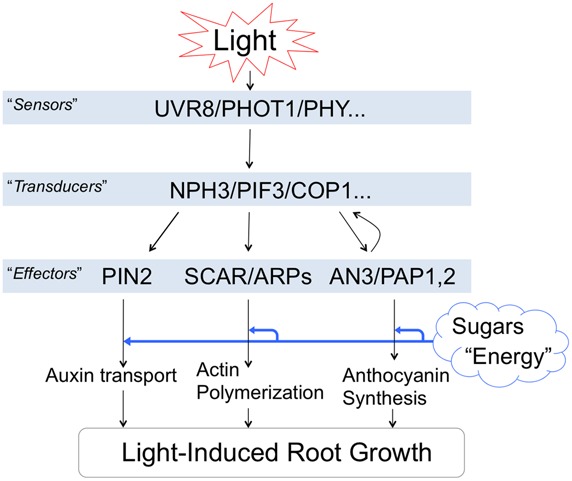
**Light-induced root growth is expensive.** Light-induced root elongation is mediated via COP1 interactions. Both SCAR and PAP mediated pathways require enough sucrose as an energy source. This figure is based on following references: Dyachok et al. (2011), Wan et al. (2012), Bai et al. (2014), Fasano et al. (2014), and Meng (2014).

It is important to be aware that light-induced stimulation of root growth is changing the physiology of whole seedlings/plants. **Figure 4** summarizes light-induced root growth mediated via COP1 interactions with the actin cytoskeleton (Dyachok et al., 2011) and anthocyanin biosynthesis (Meng, 2014). Both SCAR and PAP mediated pathways require sucrose as energy source (Kurata and Yamamoto, 1997; Kircher and Schopfer, 2012; Maier et al., 2013; Meng, 2014). Roots lacking ANGUSTIFOLIA3 (AN3) transcription coactivator have reduced anthocyanin levels and longer roots in light than WT roots (Meng, 2014). In contrast, the *an3* mutant roots are shorter that WT roots when grown in darkness. Interestingly, AN3 binds to COP1 promoter to inhibit the light-induced root elongation (Meng, 2014). Besides anthocyanins, light-exposed roots show also rather dramatic increase of phenylpropanoid metabolism, inducing not only flavonoids but also monolignol glucosides (Hemm et al., 2004). Possible impacts of light on the Casparian bands formation in endodermis can be expected and easily tested.

Light-induced actin polymerization, auxin polar transport, root growth and anthocyanin biosynthesis are energetically demanding processes. The increased demand of sucrose for the root photomorphogenesis (Costigan et al., 2011; Warnasooriya and Montgomery, 2011; Yokawa et al., 2013) is going at the expense of other potential sinks of the plant body (Kurata and Yamamoto, 1997; Yazdanbakhsh et al., 2011; Kircher and Schopfer, 2012). Moreover, salt stress perceived locally at the root apex is rapidly spread throughout the plant body via systemic signaling (Choi et al., 2014; Gilroy et al., 2014). Therefore, it is important to be aware that illumination of roots has rather dramatic consequences for the whole seedling’s physiology and morphology.

## CONCLUSIONS AND PERSPECTIVES

Our results using salt stress reveal that light exposed roots show different behavior and responses under salt stress. One possible scenario, based on reports that illuminated roots enhance strigolactone levels (Koltai et al., 2011) and strigolactone increases drought and salt tolerance of roots (Ha et al., 2014), is that the light-exposed roots are less sensitive to salt stress. Whereas the dark-grown roots show halotropism (salt avoidance tropism) by growing around the salt-enriched agar parts, the light-exposed roots grow into the salt-enriched agar if NaCl levels are not too high (**Tables 1 and 2**). As light-induced strigolactone levels modify also root responses to low phosphate (Mayzlish-Gati et al., 2012), one can propose that exposure of *Arabidopsis* roots to light is modifying their whole physiology. In fact, our preliminary data reveal that *Arabidopsis* roots exposed to light show changes the sensitivity also to the aluminum toxicity (Kagenishi et al., in preparation). In addition, IAA levels in light exposed (1 h) maize roots were more than doubled compared to control levels (Yokawa et al., in preparation). It is intriguing that enhanced strigolactone levels resemble light-induced effects on roots. It was shown that light impacts on the actin cytoskeleton, also increased abundance and recycling of PIN2 auxin transporter (Laxmi et al., 2008; Dyachok et al., 2011; Wan et al., 2012; Pandya-Kumar et al., 2014). Moreover, salt stress, drought stress, cold stress, alkaline stress, aluminum toxicity; all these challenges target the PIN2 auxin transporter which is expressed in the root apex transition zone (discussed in Baluška et al., 2010; Baluška and Mancuso, 2013). In order to avoid light exposure of roots, we recommend using of the partially darkened Petri dishes (Yokawa et al., 2011, 2013; Xu et al., 2013). This is important as stressing roots with light is modifying not only root responses to diverse stresses but also the overall physiology of such seedlings. For example, it has been reported that circadian rhythms in *Arabidopsis* roots are governed by the shoot part (James et al., 2008). Importantly in this respect, ROS signaling and homeostasis was shown to be a major modulator of circadian rhythms both in prokaryotes and eukaryotes (Edgar et al., 2012; Lai et al., 2012; Stangherlin and Reddy, 2013). It is possible that seedlings will change even their circadian clock when roots are illuminated.

**Table 2:** *Arabidopsis* root lengths in different salt and light conditions.

	Mock	100 mM	200 mM	300 mM
Dark	10.2 ± 2.9 mm	11.6 ± 2.6 mm	9.3 ± 1.8 mm	10.4 ± 2.4 mm
Light	21.1 ± 4.4 mm	20.5 ± 2.7 mm	15.9 ± 2.9 mm	14.5 ± 3.1 mm

*Root lengths were measured after 3-days-incubation on NaCl treatment plates. 20–30 roots in each plate were measured and standard deviations are presented next to the average length (mm).*

Although plant roots are heterotrophic plant organs, they require active suppression of photosynthetic gene expression. Tyrosylprotein sulfotransferase (TPST) protein HPS7 emerged recently as crucial player controlling this active suppression (Kang et al., 2014). Interestingly enough, *hsp7* mutant roots show enhanced photosynthesis-related effects under phosphate deficiency stress. Previous studies identified Golden-Like transcription factors GLK1 and GLK2 involved in activation of photosynthetic genes in roots (Waters et al., 2009; Kobayashi et al., 2013). Both *hsp7* and GLK-ox mutant roots do not show stunted root growth and any other phenotypes when grown in darkness (Kang et al., 2014).

Finally, the plant physiology perspective of the light-induced root growth in *Arabidopsis* is considering the high root growth rate as a sign of optimal root growth conditions. In stark contrast, the plant neurobiology interpretation of this increased root growth rate is that the light-exposed roots are experiencing stress and that such stressed roots are trying to escape from this unfavorable situation (Yokawa et al., 2011, 2013; Xu et al., 2013). Alpi et al. (2007), critics of the plant neurobiology initiative claimed that ‘… plant neurobiology does not add to our understanding of plant physiology, plant cell biology or signaling.’ Now, some 7 years later, it is getting obvious that plants and their roots are behaviorally much more complex than envisioned in the framework of classical plant physiology (Trewavas, 2005, 2009, 2014; Trewavas and Baluška, 2011; Baluška and Mancuso, 2013; Pierik and Testerink, 2014). Illumination of the roots is common laboratory praxis ever since *Arabidopsis thaliana* was introduced as model organism to plant sciences. However, it is important to be aware that light exposure of roots emerges as stress factor for the laboratory grown *Arabidopsis* seedlings. This is clear example for the usefulness of viewing plants as actively living and sensitive organisms solving their own plant-specific problems in intelligent manner.

## Conflict of Interest Statement

The authors declare that the research was conducted in the absence of any commercial or financial relationships that could be construed as a potential conflict of interest.
